# Transforming Growth Factor-β1 Induced Epithelial Mesenchymal Transition is blocked by a chemical antagonist of translation factor eIF4E

**DOI:** 10.1038/srep18233

**Published:** 2015-12-18

**Authors:** K. A. Smith, B. Zhou, S. Avdulov, A. Benyumov, M. Peterson, Y. Liu, A. Okon, P. Hergert, J. Braziunas, C. R. Wagner, Z. Borok, P. B. Bitterman

**Affiliations:** 1 Department of Medicine, Division of Pulmonary, Allergy, Critical Care and Sleep Medicine, University of Minnesota, Minneapolis, MN 55455 USA; 2 Department of Medicine, Division of Pulmonary, Critical Care and Sleep Medicine, Will Rogers Institute Pulmonary Research Center, University of Southern California, Los Angeles, CA USA; 3 Department of Medicinal Chemistry, University of Minnesota, Minneapolis, MN 55455 USA; 4 Department of Pharmacology, University of Minnesota, Minneapolis, MN 55455 USA

## Abstract

The epithelial to mesenchymal transition (EMT) imparts disease-defining properties to epithelial cells in cancer and organ fibrosis. Prior studies identify EMT control points at the level of transcription and translation, and indicate that activation of translation initiation factor 4E (eIF4E) is involved in the mechanisms coordinating these two levels of control. Here we show that 4Ei-1, a specific chemical antagonist of the eIF4E-mRNA cap interaction, potently inhibits transforming growth factor beta 1 (TGF-β1) mediated EMT in lung epithelial cells. Upon treatment with TGF-β1, we observed a rapid recruitment of Snail1 mRNA into the actively translated polysome pool accompanied by accumulation of the EMT transcription factor Snail1 in the nucleus. 4Ei-1 blocks ribosome recruitment to the Snail1 transcript thereby preventing accumulation of the Snail1 protein in the nucleus. Our findings establish an obligatory role for upstream translational control of downstream Snail1-mediated transcriptional events in TGF-β1 induced EMT, and provide proof of concept for efforts to pharmacologically modulate the eIF4E-cap interaction as a means to inhibit pathological EMT in the setting of cancer and organ fibrosis.

The epithelial to mesenchymal transition (EMT) is an integral step in gastrulation and organogenesis during development^1^. Triggered by a set of growth factors and morphogens including members of the transforming growth factor beta 1 (TGF-β1) super-family, EMT enables once sessile, interconnected epithelial cells to lose their apical-basal polarity, detach from one another and migrate to new locations throughout the embryo. Although intensively studied because of its centrality in the life cycle of all metazoans, interest in the EMT has expanded well beyond the realm of developmental biology. This more broad disciplinary attention has been generated by studies revealing components of the EMT developmental program in postnatal cells in at least two major categories of disease: metastatic cancer and tissue fibrosis^2^. This has led to classification of the EMT into 3 types: type 1 occurring in development, type 2 observed in tissue fibrosis and wound healing, and type 3 seen in cancer as part of the metastatic program^3^.

The EMT is orchestrated by a precisely choreographed expression of transcription factors including Snail, Twist, Slug, FoxC2, Sox4 and Zeb^4^ that repress E-cadherin expression and polarity-related genes, activate genes encoding the motility machinery and enzymes enabling invasion through connective tissue barriers, and initiate the characteristic morphological changes^5^,^6^. Although transcriptional control of EMT is well established, available evidence also highlights the importance of post-transcriptional events in the process^7^,^8^. These include RNA binding proteins governing the splicing of key EMT-related transcripts^9^,^10^,^11^,^12^,^13^ as well as mRNA export, turnover, localization and translation^2^; microRNAs targeting EMT transcription factors as well as both epithelial and mesenchymal determinants^14^,^15^; DNA methylation stabilizing the mesenchymal phenotype after EMT; the Y-box protein 1 mediated switch from cap-dependent to cap-independent translation of Snail and Zeb in Ras transformed cells^7^; the Smad4-mediated transcriptional activation of the translational repressor 4E-BP1^16^; the Akt 2-mediated relief of translational repression by RNA-binding proteins associated with the 3′-UTR of EMT transcripts^9^; and TGF-β1 mediated phosphorylation of translation factor eIF4E^17^.

The robustness of the EMT circuitry affords advantages to the developing embryo by ensuring that organogenesis and neural connections will proceed unabated by the exigencies of environmental stress; however, this robustness makes attempts to control the EMT challenging. Conceptually, efforts to intercept the EMT by interfering with apical steps such as ligand-receptor binding and upstream signal transduction, or antagonizing intermediate steps including the binding of transcription factors or microRNAs to their targets may be limited by parallel circuits that can circumnavigate the putative therapeutic block as well as by off-target effects. More appealing would be an agent that can modulate an essential downstream step in the EMT.

Whether initiated by peptide morphogens (e.g. TGF-β1, Wnt, BMP), matrix (collagens, hyaluronan) or oncogenic Ras, a feature shared by many forms of EMT is activation of signaling cascades converging on Akt2^18^. In response to Akt2 activation, three translationally controlled EMT-related reactions are initiated. One reaction is phosphorylation of the RNA binding protein hnRNPE1. In the hypophosphorylated state, hnRNPE1 binds tightly to the 3′-UTR of the mRNAs encoding two key EMT-drivers, Dab2 and ILEI, thereby repressing their translation. Upon phosphorylation, hnRNPE1 dissociates from these transcripts relieving translational repression^19^. The second reaction is phosphorylation of mTORC1, which in turn phosphorylates members of the 4E-BP translational repressor family^20^,^21^. This results in activation of the cap-dependent translation initiation complex, eIF4F. Once liberated from restraint by the 4E-BPs, eIF4F activates the translation of ILEI and other key EMT drivers^22^,^23^. The third reaction is phosphorylation of eIF4E itself on serine 209 by the Map Kinase-Interacting Kinases, which mediate eIF4E-dependent tumorigenicity^24^.

Based on this sequence of events, one prediction is that blocking activation of eIF4F-mediated translation should antagonize the EMT. Gain of eIF4F function experiments in two model organisms (*Xenopus laevis* and *Danio rerio*) have been conducted by over expressing its rate- limiting component, eIF4E, in embryonic ectodermal explants^25^,^26^,^27^. The results of these studies indicate that over expressed eIF4E is sufficient to trigger the EMT and that a highly specific eIF4E antagonist, 4Ei-1, blocks the process with no discernible toxicity^26^,^27^. However, there are no studies that directly address this issue in post-natal mammalian cells. In this report, we examine the role of eIF4F-mediated translational control in lung epithelial cell EMT. Our data indicate that lung epithelial cells can be induced to undergo the EMT by over expression of eIF4E and that TGF-β1 induced EMT can be interdicted by genetic and pharmacological interventions antagonizing eIF4E. Our data indicate that this pharmacological inhibition of TGF-β1-induced EMT is accompanied by a profound and selective suppression of ribosome recruitment to the Snail1 mRNA preventing accumulation of the Snail1 transcription factor in the nucleus. These results identify an essential, targetable step in TGF-β1-induced mammalian epithelial cell EMT that can be explored as an approach to controlling the process in cancer and fibrosis.

## Methods

### Cell lines and cell culture

RLE-6TN cells (ATCC, Manassas, VA) were cultivated in growth media [Ham’s F-12 medium (#11765-054, Gibco, Life Technologies, Grand Island, NY) supplemented with 10 mM HEPES (pH 7.2), 0.78 mM L-glutamine, 10% fetal bovine serum, 100 I.U./ml penicillin, 100 μg/ml streptomycin and 0.25 μg/ml amphotericin B]^23^. All procedures involving animals were approved by the Institutional Animal Care and Use Committee of the University of Southern California. All work was performed in accordance with the approved guidelines. Freshly isolated rat type II epithelial (AT2) cells were grown in serum-free media (MDSF) on polycarbonate filters as described^28^. For the first 48 hours, media were supplemented with 100 μg/ml *cis*-4-hydroxy-L-proline (cis-OH-proline; Sigma) to selectively eliminate fibroblasts^29^.

### Lentivirus constructs

*eIF4E:* pMSCV-3HA-eIF4E-polio IRES-eGFP was digested with Bgl II and Sal I. A 2.1 kb DNA fragment containing 3HA-eIF4E-polio IRES-eGFP was isolated by DNA agarose gel electrophoresis. pEF1α-eGFP was digested with BamHI and Sal I to remove eGFP and dephosphorylated with calf intestinal phosphatase. The 2.1 kb Bgl II/Sal I fragment was cloned into pEF1α-eGFP Bam HI/Sal I to make pEF1α-3HA-eIF4E-polio IRES-eGFP (pKAS86-12).

*4E-BP1-TTAA:* pMSCV-3HA-4E BP1-TTAA-polio IRES-eGFP was digested with Xho I and Sal I. A 2 kb fragment containing 3HA-4E BP1-TTAA-polio IRES-eGFP was isolated by DNA agarose gel electrophoresis. The 2kb Xho I/Sal I fragment was fill-in blunted with T4 DNA polymerase and cloned into pEF1α-eGFP digested with HincII/EcoRV to remove eGFP and dephosphorylated with calf intestinal phosphatase to make pEF1α-3HA-4E BP1-TTAA-polio IRES-eGFP (pKAS104-2).

### Lentivirus preparation

The infection mixture (500 μl), consisting of construct [12 μg pEF1α-3HA-eIF4E-polio IRES-eGFP (pKAS86-12), pEF1α-3HA-polio IRES-eGFP, pEF1α-3HA-4E BP1-TTAA-polio IRES-eGFP (pKAS104-2), pLKO.1 eif4E shRNA (TRCN0000077474, Open Biosystems), or control shRNA (SHC002, Sigma)], 8 μg pCMVΔR8.91, 3.5 μg pMD.G, 69 μl 2 M CaCl_2_ and H_2_O, was mixed with 500 μl warm HBS (220 mM NaCl, 60 mM HEPES and 1.5 mM Na_2_HPO_4_; pH 7.2), added drop-wise to HEK293T cells plated on 100 mm culture dishes and incubated at 37 °C overnight. Media was removed and replaced with 10 ml of DMEM (D5671, Sigma) with 10% fetal bovine serum (FBS), glutamine (2.5 mM), HEPES (20 mM), streptomycin (200 U), penicillin (200 U) and 10 mM sodium butyrate. Virus was harvested 48 h after transfection, filtered through 0.45 μm filters, concentrated with PEG-it virus precipitation solution (System Biosciences, Mountain View, CA.) and titered with a HIV p24 ELISA (Cell Biolabs, San Diego, CA.).

### Immunoblot analysis

Cell extracts were evaluated by immunoblot as previously described^30^. Primary antibodies used included: mouse anti-α-smooth muscle actin (α-SMA) (A5228, Sigma, St. Louis, MO), mouse anti-eIF4E (#610270, BD Biosciences, San Jose, CA), rat anti-HA (#11867423001, Roche, Indianapolis, IN), mouse anti-vimentin (V2258, Sigma), mouse anti-β-actin (#ab6276, Abcam, Cambridge, MA), mouse anti-GAPDH (#AM4300, Applied Biosystems, Austin, TX) and rabbit anti-lamin A/C (#SC20681, Santa Cruz Biotechnology, Santa Cruz, CA). Signal was detected with enhanced chemiluminescence (ECL, Pierce, Rockford, IL) and images were analyzed using a FluorChem imager (Alpha Innotech, San Leandro, CA).

### Immunofluorescence microscopy

Rat AT2 cells grown on filters were fixed with 4% paraformaldehyde for 10 min at RT, followed by incubation with 0.3% Triton X-100 for 10 min at RT. Cells were incubated with anti-α-SMA (#A2547,1:500; Sigma), anti-E-cadherin (#610181, 1:150; BD Biosciences Pharmingen, San Diego, CA), or anti-ZO-1 (#40-2200, 1:200; Life Technologies, Grand Island, NY) antibodies at 4 °C overnight, followed by incubation with a biotinylated anti-mouse or anti-rabbit secondary antibody (Vector Laboratories, Burlingame, CA). Signal was amplified with avidin conjugated to Texas-Red (Vector). F-actin staining was carried out as previously described^23^. Images were viewed with a NIKON Eclipse microscope equipped with a QImaging Retica 200R charge-coupled-device camera (QImaging, Surrey, BC, Canada) and acquired with the NIS-Elements BR program (NIKON). RLE-6TN cells grown on poly-L-lysine-coated coverslips were fixed with 4% paraformaldehyde for 20 min, washed with PBS, treated with 0.05% Tween 20 at RT, processed using heat-induced epitope retrieval (30 min at 98 °C in citrate buffer) and immunostained with anti-Snail1 (#ab53519, 1:100; Abcam, Cambridge, MA) antibody. Reactivity was detected using a donkey anti-goat secondary antibody conjugated with Alexa Fluor 594 (#705-585-147, 1:500; Jackson ImmunoResearch Laboratories, West Grove, PA). Images were collected from interphase cells on a Zeiss Axiovert 200M confocal microscope using AxioVision (Release 4.7) imaging capabilities.

### Induction of the epithelial to mesenchymal transition

TGF-β1 was suspended in 4 mM HCl containing 1 mg/ml BSA (vehicle) to make a stock solution (2.5 μg/ml). The TGF-β1 stock solution or vehicle were added at 1 μl per 1 ml to the medium.

Rat AT2 cells were treated with vehicle control or TGF-β1 (2.5 ng/ml) beginning on day 3. Cells were lysed in 2% SDS buffer (62.5 mM Tris-HCl, 2% SDS and 10% glycerol) for immunoblot analysis after 6 days of TGF-β1 treatment or cultures were continued until day 14 and fixed in 4% paraformaldehyde (PFA) for immunostaining.

### Genetic gain and loss of eIF4E function

*Ectopic over expression of eIF4E in AT2 cells*: Primary rat lung alveolar epithelial cells (AEC) were transduced with lentivirus expressing eIF4E (pEF1α-HA-eIF4E-IRES-eGFP) or control (pEF1α-eGFP) (MOI = 10) using polybrene (final concentration 8 μg/ml) on day 2 of culture. On day 3 of culture, treatment with vehicle control or TGF-β1 (2.5 ng/ml) was initiated. Cell extracts were prepared for immunoblot analysis after 7 days of transduction or cultures were continued until day 14 and fixed in 4% PFA for immunostaining.

*shRNA knockdown of eIF4E*: RLE-6TN cells (4 × 10^4^) were seeded into 24 well dishes, and transduced with lentivirus expressing eIF4E shRNA (TRCN0000077474, ThermoForma/Open Biosystems) or non-silencing shRNA (SHC002, Sigma) on day 1 (MOI = 1), followed by initiation of TGF-β1 (2.5 ng/ml) treatment on day 2. After 2 days of TGF-β1 treatment (day 4), cell extracts were prepared for immunoblot analysis of α-SMA expression. A similar procedure was used for inhibition of eIF-4E in RLE-6TN cells by transducing cells with virus expressing constitutively active 4E-BP1 (pEF1α-3HA-4EBP1 TTAA-IRES-GFP) or control virus (pEF1α-eGFP).

### Pharmacological antagonism of eIF4E function

AT2 cells cultured on polycarbonate filters coated with collagen-1 at a density of 0.5 × 10^6^/cm^2^ were treated with TGF-β1 (2.5-3.5 ng/ml) ± 4Ei-1 (500 μM) (vehicle was used as control) beginning on day 2 after seeding. Media were changed every other day and cells were harvested for Western blotting and fixed for immunostaining at day 14.

For Snail1 intercellular localization, RLE-6TN cells were seeded onto 12 mm poly-L-lysine coated coverslips in 24 well cell culture plates (80,000 cells per well) in medium containing 10% FBS and allowed to adhere overnight. Approximately 12 h later, medium in each well was removed and replaced with 0.5 ml of medium containing 0.6% FBS. After 4 h of culture in 0.6% FBS, either 4Ei-1 (100 μM) or vehicle (sterile water) was added to the culture medium and the cells incubated for another 4h. TGF-β1 (2.5 ng/ml) or vehicle was added to the culture medium and the cells incubated for an additional 6 h before fixing.

### Migration Assay

RLE-6TN cells were seeded on cell culture inserts (24 well, 8 μm pore size; BD Falcon) at ~7–9 × 10^3^ cells per well with input cell numbers adjusted to account for the growth inhibitory effect of TGF-β1. Inserts were placed in 24 well dishes in growth medium and incubated overnight (37 °C, 5% CO_2_). Approximately 18 h after seeding, medium was replaced with growth medium on the bottom of the insert and Ham F12 + 2% FBS on top of the insert. Treatment with vehicle control or TGF-β1 (2.5 ng/ml) ± 4Ei-1 (100 μm) was initiated and continued for 72 h with medium changed every 12 h to insure active TGF-β1 and 4Ei-1 was present throughout the assay in the wells receiving those treatments. Cells on the top surface of the insert membranes were removed by scraping and inserts were fixed in methanol, stained in 0.1% crystal violet and washed 3 times in H_2_O per the manufacturer’s protocol. Filters were cut from each insert with a scalpel and cells that had migrated through the filter were counted (15 fields per filter, 3 filters per condition). A migration index [migration of cells treated with TGF-β1 or (TGF-β1 + 4Ei-1)]/[migration of cells treated with vehicle control] was calculated.

### Conversion of the 4Ei-1 prodrug to biologically active 7-Benzyl GMP by RLE6TN cells

7Bn-GMP, 4Ei-1, and 7-ortho-F-Bn-GMP were synthesized as previously described^31^,^32^,^33^,^34^. The 4Ei-1 prodrug ([2-(3-Indolyl)-1-ethyl]phosphoramidic Acid 5′-(7-Benzylguanosyl) Monophosphate) is bioactivated by cellular enzyme histidine triad nucleotide binding protein (HINT) to 7-benzyl GMP. To assess whether the RLE-6TN cells converted the 4Ei-1 prodrug to biologically active 7-benzyl GMP, triplicate culture dishes, each containing 5 × 10^6^ RLE-6TN cells were incubated in growth medium +/− 500 μM 4Ei-1 for 4 h. Cells were removed from culture dishes by scraping, washed twice in phosphate-buffered saline, pelleted by centrifugation and frozen at −80 °C. To initiate the analysis, each cell pellet was suspended in a 0.5 ml mixture of methanol and 10 mM ammonium acetate (v/v = 60:40), sonicated twice, lyophilized and subjected to HPLC-ESI-MS/MS analysis as previously described^33^. Each of the triplicate biological samples was analyzed 3 times by HPLC-ESI-MS/MS giving 3 technical replicates for each biological sample.

### Polyribosome preparations and qRT-PCR

RLE-6TN cells were divided into 2 groups. One group was pre-treated for 4 h with 4Ei-1 (200 μM) and the other with vehicle. Both groups were subsequently treated for 2 h with medium containing TGF-β1 (2.5 ng/ml) in the presence or absence of 4Ei-1. Cells treated only with vehicle for 4Ei-1 (water) and TGF-β1 (1 μl per ml, 4 mM HCl containing 1 mg/ml BSA) served as controls. Total RNA and cytosolic ribosome-bound RNA (polysomes) were isolated from each cell preparation as described previously^35^. Reverse transcription of all fractions was completed using a Taqman reverse transcription kit (Roche) and qRT-PCR was performed on the total RNA and across the polysome gradient fractions for Snail1 mRNA and β-actin mRNA (control) using a Roche Lightcycler model 1.5 and Lightcycler Fast Start Master Plus SYBR reagent kit as instructed by the manufacturer. All primers were prepared by the University of Minnesota Genomics Center. Snail1 primers were as follows:

Sense = 5′-AGTTGTCTACCGACCTTGCG-3′;

Anti-sense = 5′-TGCAGCTCGCTATAGTTGGG-3′.

β-actin primers were as follows:

Sense = 5′-GATCAAGATCATTGCTCCTCCTGA -3′;

Anti-sense = 5′- ACGCAGCTCAGTAACAGTCC-3′.

Using Snail1 primers at a concentration of 500 nM, amplification was performed for 40 cycles using an annealing temperature of 60 °C and an extension time of 6 seconds. β-actin primers were used at a concentration of 500 nM and amplification was performed for 35 cycles using an annealing temperature of 60 °C and an extension time of 8 seconds. Samples were quantified at the log-linear portion of the curve using LightCycler analysis software. Amplified products were analyzed by agarose gel electrophoresis and yielded a single product of the expected size.

## Results

### Overexpression of eIF4E in primary lung epithelial cells induces the EMT

**Figure 1:**
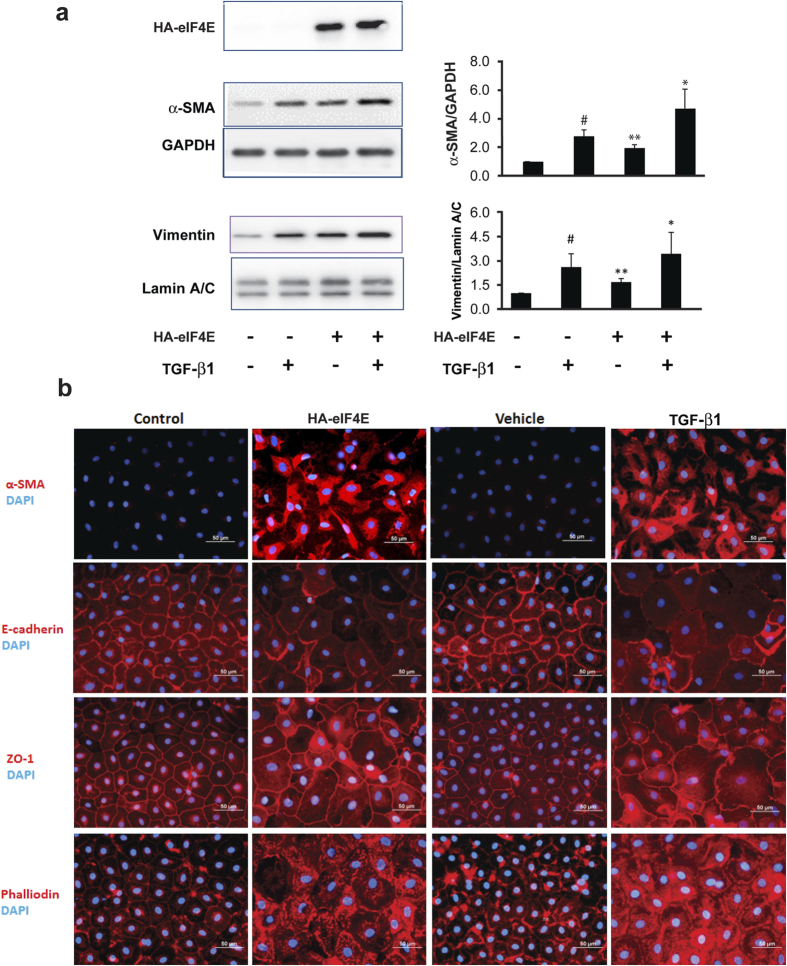
Ectopic overexpression of eIF4E in primary lung epithelial cells induces the EMT. Primary rat AT2 cells were transduced with virus expressing HA-eIF4E (pEF1α-HA-eIF4E-IRES-GFP) or a control gene (pEF1α-GFP) and treated the next day with TGF-β1 (2.5 ng/ml) or vehicle (1 μl per 1 ml 4 mM HCl containing 1 mg/ml BSA). (**a**) Cell lysates were harvested after 6 days of TGF-β1 treatment. Shown are immunoblots (left panels) probed for HA-eIF4E, α-SMA (GAPDH served as the loading control) and vimentin (Lamin A/C served as the loading control). Immunoblots shown are representative of 4 independent experiments. The mean values for all 4 replicates are shown (right panels). ^#^indicates P < 0.05 for the comparison of TGF-β1 versus vehicle; **indicates P < 0.05 for the comparison of HA-eIF4E versus vector; and * indicates P < 0.05 for the comparison of HA-eIF4E alone versus HA-eIF4E + TGF-β1. (**b**) Cells were fixed with 4% paraformaldehyde 12 days following transduction with lentivirus. Immunofluorescence is shown for α-SMA, F-actin (phalloidin), E-cadherin and ZO-1. Images shown are representative of 3 independent experiments. TGF-β1 is shown as a positive control.

When ectopically expressed in embryonic ectodermal explants, eIF4E induces the EMT^25^,^27^. To examine the effects of eIF4E on the differentiated state of post-natal lung epithelial cells, we ectopically expressed hemagglutinin-tagged eIF4E (HA-eIF4E) in rat AT2 cells (Supplementary Figure 1) and examined them for evidence of EMT. Treatment with TGF-β1 (2.5 ng/ml) served as a positive control. In cells expressing HA-eIF4E, there was an approximately 2-fold increase in the mesenchymal cytoskeleton components, α-smooth muscle actin and vimentin, a response comparable to that observed with TGF-β1 (Fig. 1a). Together, HA-eIF4E and TGF-β1 led to an even more robust induction of α-smooth muscle actin and vimentin than either alone. Morphologically, in response to either eIF4E or TGF-β1, we observed an increase of α-smooth muscle actin and F-actin with an associated loss of strict localization of membrane-associated E-cadherin and zona occludens protein 1 (ZO-1) at the perimeter of the cell (Fig. 1b). As additional validation, immunoblot analysis confirmed that both eIF4E and TGF-β1 negatively regulated E-cadherin and Claudin-18 (Supplementary Figure 2). These data indicate that similar to TGF-β1, ectopic expression of eIF4E induces biochemical and morphological changes characteristic of the EMT.

### Inhibition of eIF4E blocks TGF-β1 induced α-smooth muscle actin expression and formation of an actin filament network.

**Figure 2:**
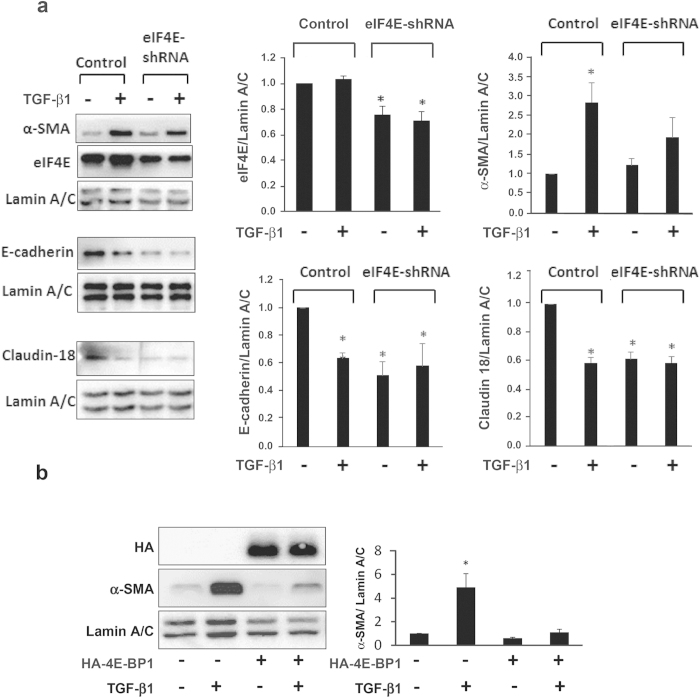
eIF4E antagonism attenuates TGF-β1-induced α-SMA expression in RLE-6TN cells. (**a**) RLE-6TN cells were seeded at a density of 40,000 in 24-well-plates, and transduced with virus expressing eIF-4E shRNA (pLKO.1 eif4E shRNA; TRCN0000077474, Open Biosystems), or control non-silencing shRNA (SHC002, Sigma) on day 1 at MOI of 2.5, followed by TGF-β (2.5 ng/ml) treatment on day 2 (vehicle (V = 1 μl per 1 ml 4 mM HCl containing 1 mg/ml BSA) as control). Protein was harvested for Western analysis of eIF-4E, α-SMA, E-cadherin and Claudin 18 expression after 2 days of treatment; Lamin A/C is the loading control. Blots shown are representative of 3 independent experiments. Densitometric analysis demonstrated that knockdown of eIF-4E significantly reduced TGF-β-induced α-SMA expression as well as blocked TGF-β-induced reductions in E-cadherin and Claudin18 expression, suggesting eIF-4E plays an important role in regulation of genes involved in TGF-β-induced EMT in AEC. *P < 0.05, represents significant difference of eIF-4E expression from control shRNA, significant difference of α-SMA expression from other conditions, and significant difference of E-cadherin and Claudin 18 expression from vehicle control (1 μl per 1 ml 4 mM HCl containing 1 mg/ml BSA). (**b**) RLE-6TN cells were transduced with virus expressing a constitutively active form of the translational repressor HA-4EBP1 (pEF1α-3HA-4EBP1-TTAA-IRES-GFP) or control (pEF1α-eGFP). The next day, cells were treated with TGF-β1 (2.5 ng/ml) or vehicle (1 μl per 1 ml 4 mM HCl containing 1 mg/ml BSA). Cell lysates were harvested after 2 days of treatment. Shown is an immunoblot (left panel) probed for α-SMA expression (Lamin A/C served as the loading control). The immunoblot shown is representative of 3 independent experiments. The mean values for all 3 replicates are shown (right panel). *indicates P < 0.05 for the comparison of TGF-β1 treated cells expressing HA-4E-BP1 to all other conditions.

The mechanisms by which TGF-β1 induces EMT include both canonical and non-canonical signaling pathways which can have opposing effects on the translational machinery. Canonical TGF-β1 signaling includes SMAD4 mediated transcriptional activation of the translational repressor 4E-BP1^16^, which inhibits the function of eIF4E. However, non-canonical signaling can lead to hyperphosphorylation of 4E-BP1 which liberates eIF4E enabling it to form an active translational complex, eIF4F^36^, and leads to phosphorylation of eIF4E^37^. To determine whether eIF4E activity was required for TGF-β1-induced EMT in respiratory epithelial cells, we antagonized eIF4E function in RLE-6TN cells using two independent genetic approaches. In the first, we introduced a shRNA targeting eIF4E mRNA (Fig. 2a) and in the second, we ectopically expressed a constitutively active form of HA-tagged 4E-BP1 to sequester the eIF4E protein (Fig. 2b; endogenous 4E-BP1 shown in Supplementary Figure 3). Negative regulation of eIF4E by either approach decreased or greatly attenuated the ability of TGF-β1 to induce α-smooth muscle actin in RLE-6TN cells; and blocked the TGF-β1-induced decrease of E-cadherin and Claudin-18 (additional markers for 4E-BP1 antagonism are shown in Supplementary Figure 4). These results directly show that the TGF-β1 mediated shift from an epithelial towards a mesenchymal phenotype is strictly dependent upon the activity of eIF4E.

### Pharmacological antagonism of eIF4E blocks TGF-β1 mediated actin reorganization and motility

We have previously described the synthesis and characterization of a phosphoramidated 7-benzyl-GMP (7-Bn-GMP) pro-drug designated 4Ei-1 that potently antagonizes the association of eIF4E with the 5′ mRNA cap^31^,^32^. 4Ei-1 is non-toxic, water soluble, membrane permeable and rapidly activated within the cell by the enzyme HINT (conversion of 4Ei-1 to bioactive 7-Bn-GMP in RLE-6TN cells shown in Supplementary Figure 5). Once activated, it negatively regulates the association of the 5′ mRNA cap with eIF4E resulting in a dose-dependent inhibition of cap-dependent translation.

**Figure 3:**
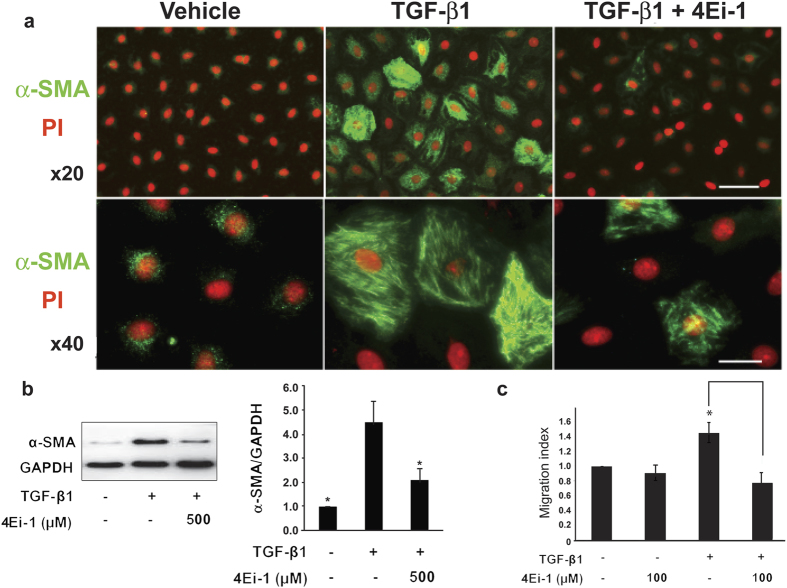
Pharmacological antagonism of eIF4E blocks TGF-β1 mediated actin reorganization and motility. Primary rat AT2 cells were seeded onto type 1 collagen coated filters at a density of 5 × 10^5^ cells/cm^2^. After 2 days, cells were treated with TGF-β1 (2.5 ng/ml) with or without 4Ei-1 (500 μM) with medium replenished every other day until day 14 when they were fixed with 4% paraformaldehyde or harvested for protein. (**a**) Shown are immunofluorescence images with anti-α-SMA antibody (green) and propidium iodide (red) to visualize nuclei; upper panel at x20 (Bar = 50 μm), lower panel at x40 (Bar = 15 μm). (**b**) Shown is an immunoblot (left panel) probed for α-SMA with GAPDH as the loading control. Immunoblot shown is representative of 4 independent experiments. The mean quantitation values for all 4 replicates are shown (right panels). *indicates P < 0.05 for the comparison of vehicle versus TGF-β1 and TGF-β1 + 4Ei-1 versus TGF-β1. **(c)** RLE-6TN cells were seeded onto cell culture inserts and treated with vehicle or TGF-β1 (2.5 ng/ml) ± 4Ei-1 (100 μM) for 72 h. Shown for each condition is the migration index [migration of cells treated with TGF-β1 or (TGF-β1 + 4Ei-1)]/[migration of cells treated with vehicle control]. *indicates P < 0.05.

When lung epithelial cells were treated with TGF-β1, we observed the expected biochemical and functional hallmarks of the EMT. These included reorganization of the actin filament network in AT2 cells (Fig. 3a); increased expression of α-smooth muscle actin (Fig. 3b); and increased motility (Fig. 3c) in RLE-6TN cells. In sharp contrast, when cells were incubated with both TGF-β1 and 4Ei-1, these changes were markedly attenuated. These data indicate that 4Ei-1 effectively blocked the acquisition of TGF-β1-induced EMT hallmarks.

### Pharmacological antagonism of eIF4E profoundly suppresses TGF-β1-mediated ribosome recruitment to the Snail1 transcript and nuclear accumulation of Snail1 protein in RLE-6TN cells

**Figure 4:**
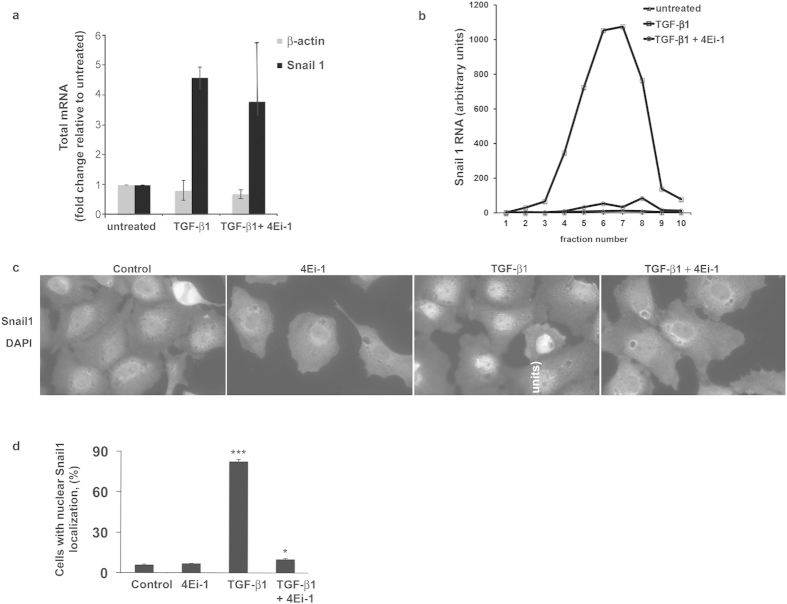
Pharmacological antagonism of eIF4E profoundly suppresses TGF-β1-mediated ribosome recruitment to the Snail1 transcript and nuclear accumulation of Snail1. Following pretreatment with 4Ei-1 (200 μM) for 4 h, or no pretreatment, RLE-6TN cells were treated with TGF-β1 (2.5 ng/ml) for 2 h, and processed for total and polysome bound RNA. (**a**) Shown are values for Snail1 mRNA and β-actin mRNA by qRT-PCR found in the total RNA samples normalized to the untreated sample. Shown are mean values for two independent experiments. (**b**) Relative Snail1 mRNA values across the 10 gradient fractions of polysome bound mRNA as analyzed by qRT-PCR. (**c**) To determine Snail1 intercellular localization, RLE-6TN cells were changed to medium with 0.6% serum. After 4 h, cells were treated with 4Ei-1 (100 μM) or vehicle (control) for an additional 4 h followed by TGF-β1 (2.5 ng/ml) or vehicle for 6h. Shown are representative images demonstrating nuclear Snail1 in TGF-β1-treated cells and cytoplasmic Snail1 in control, 4Ei-1, and TGF-β1 ± 4Ei-1-treated cells. d. Quantification of cells with nuclear Snail1: TGF-β1, p < 0.001(***), TGF-β1 + 4Ei-1, p < 0.05 (*).

In many cell systems, the transcription factor Snail1 is an important component of a TGF-β1 triggered transcriptional network that includes Snail1, Twist, Slug, FoxC2 and Zeb^30^. These transcription factors function to repress epithelial genes and activate mesenchymal genes^38^. To explore whether Snail1 functioned at the intersection of the transcriptional and translational TGF-β1 signaling network, we quantified total and polysome-associated Snail1 mRNA after TGF-β1 treatment. In accord with prior reports, we observed a very rapid, 4-fold increase in steady state Snail1 mRNA levels in response to TGF-β1 (Fig. 4a)^39^.

Translational activation can manifest in an “off-on” manner with recruitment of mRNA into the actively translated polysome pool, and as an increase in the average number of ribosomes per transcript^40^,^41^,^42^. Analysis of TGF-β1 stimulated cells showed a nearly 100-fold increase in the abundance of Snail1 mRNA in the actively translated polysome pool (Fig. 4b); with no significant change in the number of ribosomes bound per Snail1 transcript (supplementary Figure 6A and 6B)^17^. Importantly, this increase of ribosome recruitment to Snail1 mRNA was abolished by 4Ei-1 (Fig. 4b). Of note, transcriptional activation of Snail1 was relatively unaffected by 4Ei-1 with only a modest effect on the association of β-actin mRNA with polysomes (Supplementary Figure 7; global polysome tracings for all conditions shown in Supplementary Figure 8), thus excluding a non-specific toxic effect of 4Ei-1. TGF-β1 treatment resulted in nuclear accumulation of Snail1 protein and this nuclear accumulation was ablated by 4Ei-1 treatment (Fig. 4c,d). These data link cap-dependent translational activation of Snail1 by TGF-β1 with the ability of eIF4E to recruit Snail1 mRNA to polysomes and inhibit the EMT, thus revealing an obligatory role for upstream translational control of downstream transcriptional events in TGF-β1-induced EMT, and a potentially targetable step in pathological EMT.

## Discussion

Although EMT is an essential step in embryonic development, its role in wound healing, fibrosis and cancer in post-natal life has motivated efforts to control the process. Although prior studies identified EMT control points both at the level of transcription and translation, it remained to be determined whether this knowledge could be exploited therapeutically. Here we report the discovery of an obligatory role for upstream translational control of downstream transcriptional events in TGF-β1-induced lung epithelial cell EMT. Our data show that lung epithelial cells can be induced to undergo the EMT by over expression of the rate-limiting component of the cap-dependent translation initiation machinery, eIF4E, in the absence of exogenous TGF-β1, and that TGF-β1-induced EMT can be interdicted by genetic and pharmacological interventions antagonizing eIF4E. In addition, we unveiled a 2-orders of magnitude activation of ribosome recruitment to Snail1 mRNA in response to TGF-β1 that was nearly completely ablated by a pharmacological antagonist of eIF4E that blocks the EMT. Our findings disclose an essential role for translational control at the apex of the transcriptional network in TGF-β1-induced EMT that can be explored as a targetable step for controlling the process in cancer and fibrosis.

Prior studies by the Howe group definitively identified a role for translational control of the EMT effectors ILEI and Dab2 in response to TGF-β1^19^, and the Sonenberg group established a central role for eIF4E phosphorylation in TGF-β1 induced EMT^17^. However, our study is the first to show that ectopic over expression of eIF4E alone is sufficient to trigger primary mammalian epithelial cells to undergo the EMT. Our work unveils an extremely rapid, hundred-fold activation of ribosome recruitment to the apical EMT transcription factor Snail1 in response to TGF-β1, and documents that a critical threshold level of eIF4E activity is necessary both for translational activation of Snail1 and TGF-β1-induced EMT. These findings strongly support the centrality of cap-dependent translational control in post-natal mammalian EMT.

Our work builds on two prior studies of eIF4E-mediated EMT that used ectodermal embryonic explants from lower vertebrates. Nearly two decades ago, the Melton laboratory showed that ectopic eIF4E expression in *Xenopus* ectodermal explants could drive mesenchymal differentiation^25^. More recently, we reported the synthesis of a small molecule inhibitor of eIF4E that competitively antagonizes the association of the 5′ mRNA cap with the cap-binding pocket of eIF4E, and showed that it blocked eIF4E-induced EMT in zebrafish ectodermal explants^27^. Here we advance the field of eIF4E-antagonism by showing that the phosphoramidated nucleoside 4Ei-1 is rapidly bioactivated in mammalian lung epithelial cells to 7-benzyl-GMP and that it blocks TGF-β1-induced Snail1 translation and the EMT without discernible toxicity as judged by preservation of morphology, Snail1 transcription and β-actin translation. Thus, our findings provide compelling proof of concept for efforts to develop pharmacological agents that can inhibit the EMT by modulating eIF4E activity.

## Additional Information

**How to cite this article**: Smith, K. A. *et al.* Transforming Growth Factor-β1 Induced Epithelial Mesenchymal Transition is blocked by a chemical antagonist of translation factor eIF4E. *Sci. Rep.* **5**, 18233; doi: 10.1038/srep18233 (2015).

## Supplementary Material

Supplementary Information
