# Supplementary material for: Transforming Growth Factor-β1 Induced Epithelial Mesenchymal Transition is blocked by a chemical antagonist of translation factor eIF4E
Source: Sci Rep. 2015 Dec 18;5:18233. doi: 10.1038/srep18233 (PMC4683370; doi:10.1038/srep18233)
Supplement: Supplementary Information [file srep18233-s1.pdf]

SUPPLEMENTARY MATERIALS

Title: Transforming Growth Factor-β1 Induced Epithelial Mesenchymal Transition is blocked by a chemical antagonist of translation factor eIF4E

K.A. Smith [1*], B. Zhou [2*], S. Avdulov [1], A. Benyumov [1], M. Peterson [1], Y. Liu [2], A. Okon [3], P.Hergert [1], J. Braziunas [4], C. R. Wagner [3], Z. Borok [2] and P. B. Bitterman [1**.]

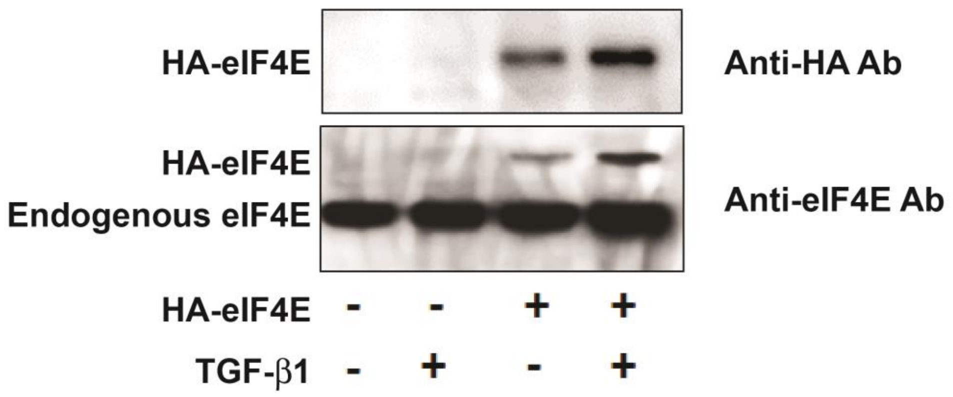

Supplementary Figure 1*.* Ectopic overexpression of eIF4E in primary lung epithelial cells. AT2 cells were transduced with virus expressing HA-eIF4E (pEF1α-HA-eIF4E-IRES-GFP) or control (pEF1α-GFP) and treated the following day with TGF-β1 (2.5 ng/ml) or vehicle (1 µl per 1 ml 4 mM HCl containing 1 mg/ml BSA). Cell lysates were harvested after 6 days of TGF-β1 treatment. Western blotting (WB) shows overexpression of HA-eIF4E in virus- transduced samples using both anti-HA-eIF4E and anti-HA antibodies.

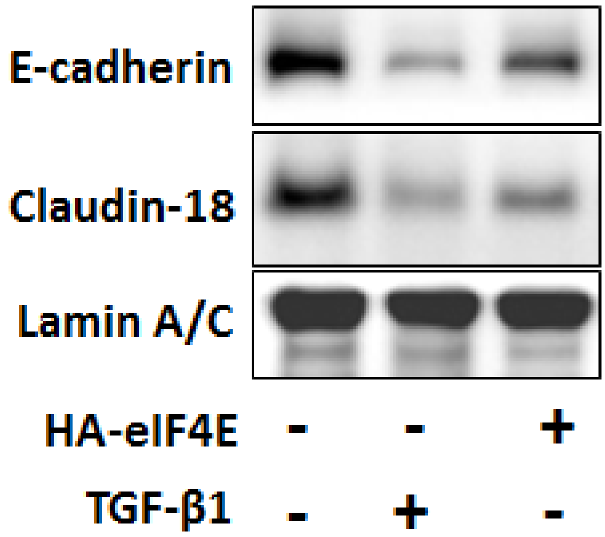

Supplementary Figure 2. Primary rat AT2 cells were transduced with virus expressing HA-eIF4E (pEF1α-HA-IRES-GFP) or control (pEF1α-GFP) and treated the next day with TGF-β1 (2.5 ng/ml) or vehicle (1μl per ml 4 mM HCl containing 1 mg/ml BSA). Cell lysates were harvested after 6 days of TGF-β1 treatment. Shown is an immunoblot probed for E-cadherin and claudin-18. Lamin A/C served as a loading control.

HA-eIF4E-BP1 (TTAA) overexpression in RLE 6TN cells

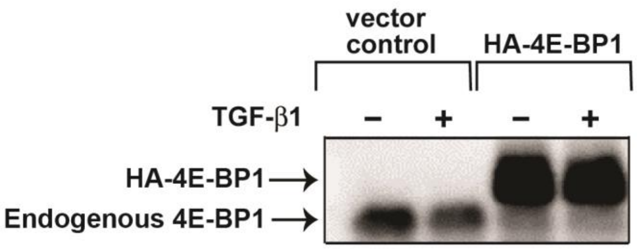

Supplementary Figure 3*.* Ectopic overexpression of HA-eIF4E-BP1 (TTAA) in primary lung epithelial cells.

AT2 cells were transduced with virus expressing HA-4E-BP1(TTAA) (pEF1α-HA-4E-BP1{TTAA}-IRES-GFP) or control (pEF1α-GFP) and treated the following day with TGF-β1 (2.5 ng/ml) or vehicle (1 µl per 1 ml 4 mM HCl containing 1 mg/ml BSA). Cell lysates were harvested after 6 days of TGF-β1 treatment. Western blotting (WB) shows overexpression of HA-4E-BP1 in virus- transduced samples using anti-4E-BP1 antibody.

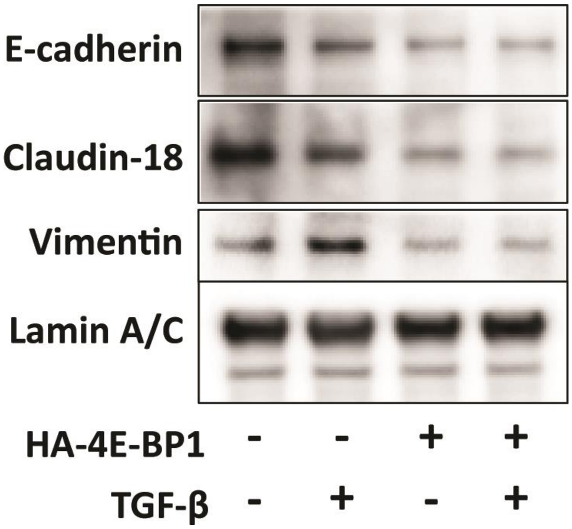

Supplementary Figure 4. RLE-6TN cells were transduced with virus expressing a constitutively active form of the translational repressor HA-4E-BP1 (pEF1α-HA-4E-BP1(TTAA)-IRES-GFP) or control (pEF1α-GFP). The next day, cells were treated with TGF-β1 (2.5 ng/ml) or vehicle (1 µl per 1 ml 4 mM HCl containing 1 mg/ml BSA). Cell lysates were harvested after 2 days of treatment. Shown is an immunoblot probed for E-cadherin, claudin-18, and vimentin. Lamin A/C served as a loading control.

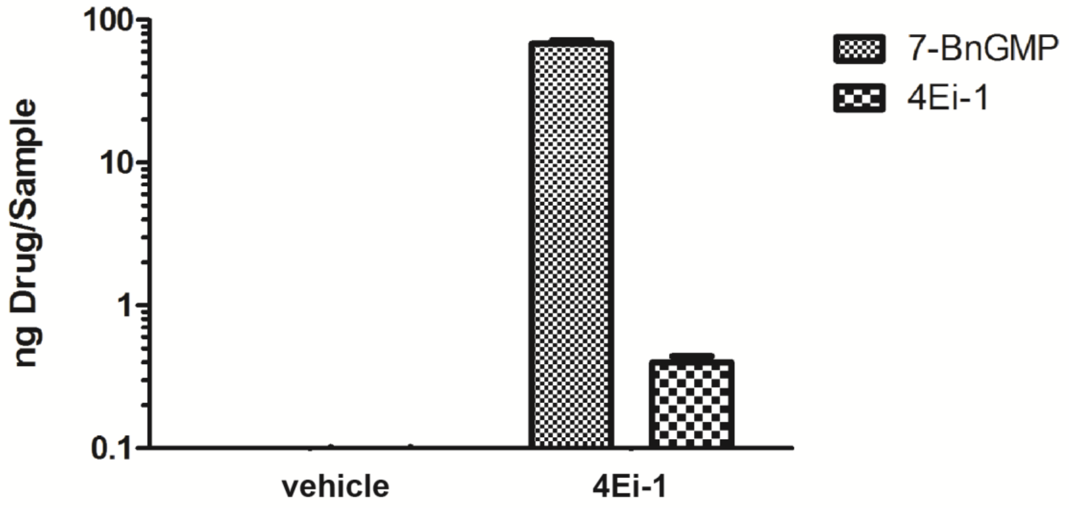

Supplementary Figure 5. 4Ei-1 is bioactivated to 7-Bn-GMP by RLE6TN cells. Shown are the amounts of 4Ei-1 (prodrug) and 7-Bn-GMP (active form) within RLE-6TN cells after incubation with 4Ei-1 (500 μM in growth medium) for 4 h.

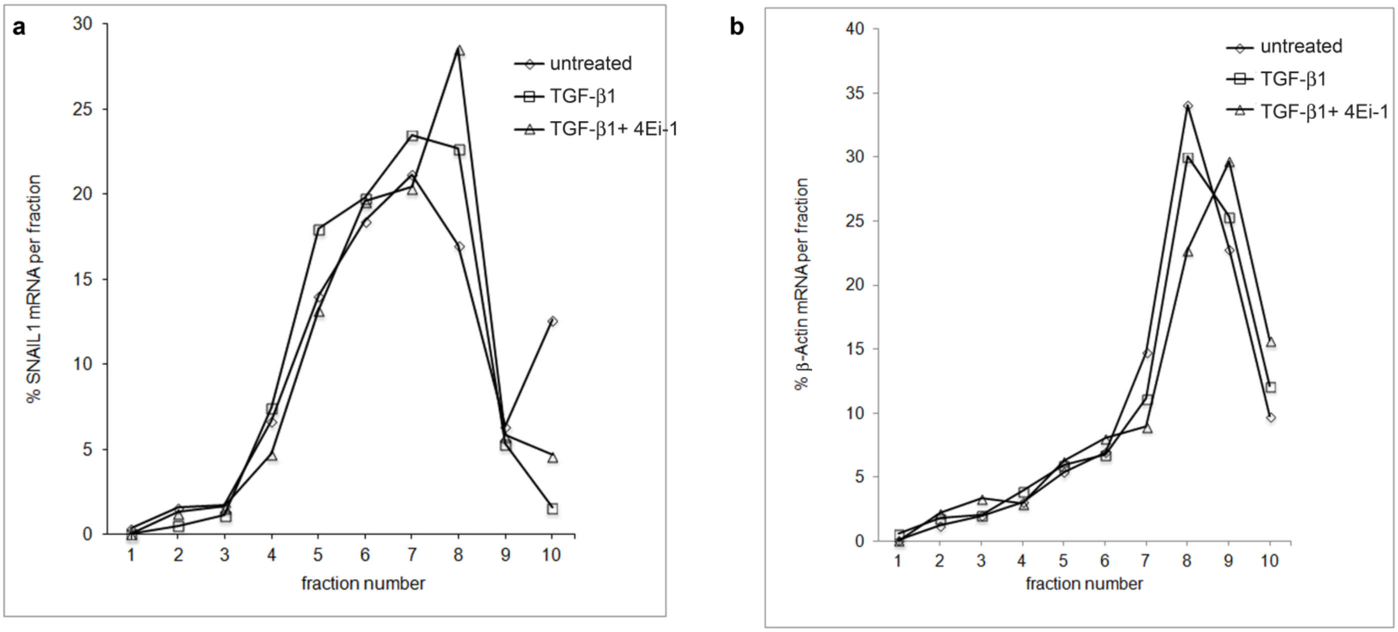

Supplementary Figure 6. TGF-β1 does not significantly alter the ribosome loading profile of Snail1 or β-actin mRNA. RLE-6TN cells were treated with TGF-β1 (2.5 ng/ml) for 2h with or without 4Ei-1 (200 μM) pretreatment for 4 h and processed for polysome analysis. (**a)** Shown are values for Snail1 and (**b)** β-actin mRNA abundance in each fraction obtained by qRT-PCR expressed as % mRNA per fraction. Values depicted are the averages of 2 independent sets of experiment. The weighted average fraction values for Snail1 were nearly identical across all 3 conditions: untreated = 6.8, TGF-β = 6.5, TGF-β +4Ei-1 = 6.8; as were the weighted averages for β-actin: 7.7, 7.6, and 7.8 respectively.

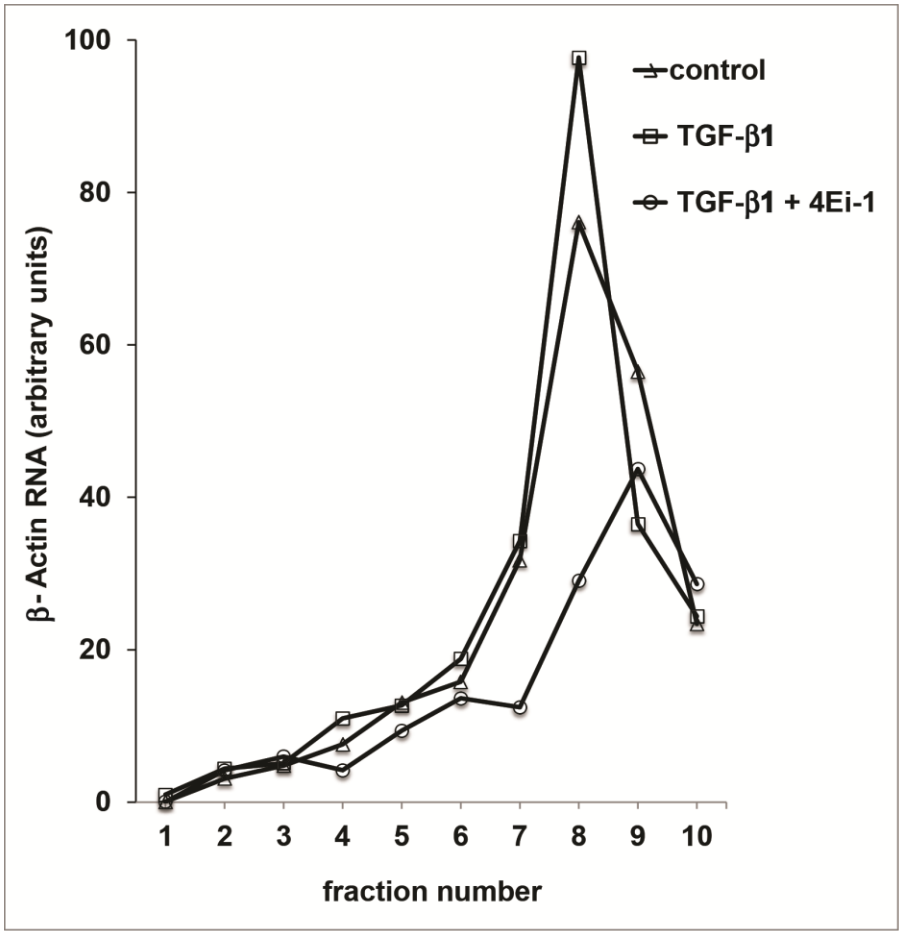

Supplementary Figure 7. Effect of 4Ei-1 on β-actin mRNA abundance in the polysome pool. RLE-6TN cells were treated with TGF-β1 (2.5 ng/ml) for 2h with or without 4Ei-1 (200 μM) pretreatment for 4 h and processed for polysome bound RNA. Shown are normalized values for β-actin mRNA abundance obtained by qRT-PCR under the indicated conditions across the 10 gradient fractions analyzed.

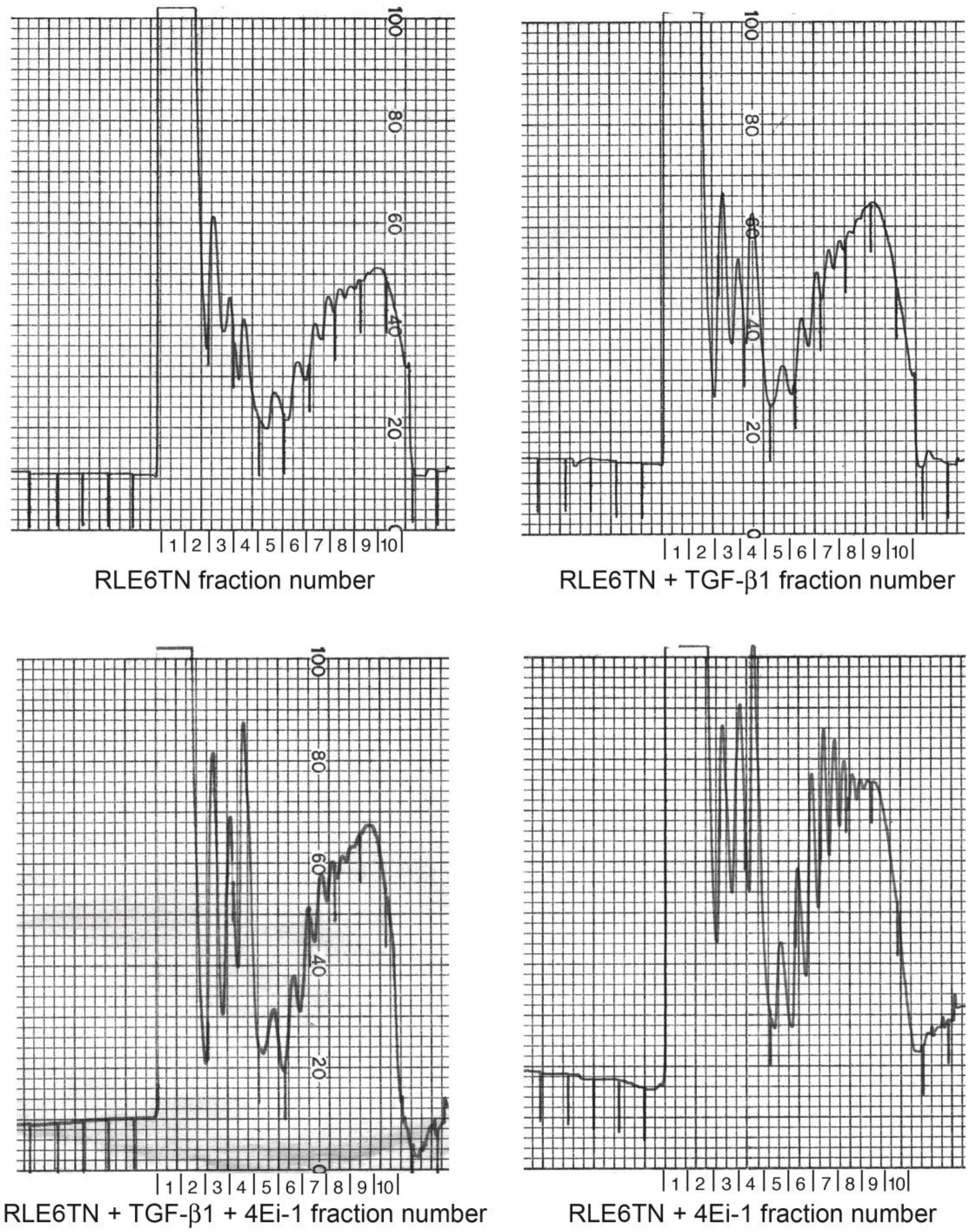

Supplementary Figure 8. RLE-6TN cell polysome tracings. Shown are representative polyribosome tracings for RLE-6TN cells from the 4 treatment conditions: no treatment, TGF-β1 (2.5 ng/ml, 2h), 4Ei-1 (200μM, 4 h) or 4Ei-1 (200μM, 4 h) then TGF-β1 (2.5 ng/ml, 2h).
